# Predictors of long-term (≥ 6 months) antipsychotic polypharmacy prescribing in secondary mental healthcare

**DOI:** 10.1016/j.schres.2016.04.010

**Published:** 2016-07

**Authors:** Giouliana Kadra, Robert Stewart, Hitesh Shetty, Johnny Downs, James H. MacCabe, David Taylor, Richard D. Hayes

**Affiliations:** a King's College London, Institute of Psychiatry, Psychology and Neuroscience, London, UK; b South London and Maudsley NHS Foundation Trust, London, UK

**Keywords:** Antipsychotic polypharmacy, Predictors, Service use, Clinical symptoms, Socio-demographic, Socioeconomic

## Abstract

**Introduction:**

The predictors of long-term antipsychotic polypharmacy (APP) initiation are poorly understood. Existing research has been hampered by residual confounding, failure to exclude cross-titration, and difficulties in separating the timing of predictors and APP administration.

**Materials and methods:**

Using data from the South London and Maudsley (SLaM) case register, we identified all adult patients with serious mental illness (SMI) who were receiving care between 1st July 2011 and 30th June 2012. Exposures measured between 1st July and 31st December 2011 included socio-demographic, socioeconomic, clinical and service use characteristics. We then determined if long-term APP (six or more months) had been initiated between 1st January and 30th June 2012. Multivariable logistic regression models, adjusted for socio-demographic and socioeconomic factors, were built to investigate the associations between the above factors and the initiation of long-term APP.

**Results:**

We identified 6857 adults with SMI receiving SLaM care, of whom 115 (1.7%) were newly prescribed long-term APP. In the adjusted models, predictors of long-term APP initiation included: symptoms (severity of hallucinations and/or delusions), previous treatments (clozapine and long-acting injectable antipsychotic agents), service use (more contact with outpatient services, community treatment order receipt), social factors (higher area-level deprivation, homelessness) and socio-demographic status (younger age, not in a relationship).

**Conclusion:**

Our findings highlight that certain patient groups are at an increased risk for long-term APP initiation. Identifying these groups earlier in their treatment could encourage clinicians to employ a broader range of interventions in addition to pharmacotherapy to reduce the risk of APP prescribing.

## 1 Introduction

Antipsychotic polypharmacy (APP; the concomitant administration of two or more antipsychotics) remains common practice in treatment of serious mental illnesses (SMI). Its prevalence is estimated to vary between 10 and 30% (Freudenreich and Goff, 2002, Gallego et al., 2012), despite current guidelines recommending against APP use, except during clozapine augmentation (APA, 2004, Lochmann van Bennekom et al., 2013, NICE and NCCMH, 2013), and evidence of associations with increased mortality (Ganguly et al., 2004, Joukama et al., 2006, Waddington et al., 1998) and physical health problems (including metabolic and cardiovascular disorders) (Ganguly et al., 2004, Raedler, 2010). Examining factors that may predict APP prescribing is key to understanding its continued use.

To date, male gender (Ganguly et al., 2004, Kreyenbuhl et al., 2007a, Kreyenbuhl et al., 2007b, Morrato et al., 2007, Suokas et al., 2012), and younger age (Kreyenbuhl et al., 2007a, Kreyenbuhl et al., 2007b, Morrato et al., 2007, Suokas et al., 2012) have been found to be associated with APP, but there has been a lack of information on socioeconomic factors (Barbui et al., 2006). APP has been found to be associated with more frequent previous hospital admissions (Faries et al., 2005, Ganguly et al., 2004, Kreyenbuhl et al., 2007a, Kreyenbuhl et al., 2007b, Morrato et al., 2007), longer duration of previous admissions (Suokas et al., 2012), higher number of previous outpatient contacts (Ganguly et al., 2004, Kreyenbuhl et al., 2007a, Kreyenbuhl et al., 2007b) and previous antipsychotic medication use (Barbui et al., 2006, Ganguly et al., 2004). Findings regarding the role of clinical symptoms in APP prescribing have been inconsistent (Barbui et al., 2006, Biancosino et al., 2005, Centorrino et al., 2005).

Previous research has examined APP of varying duration (Broekema et al., 2007, Clark et al., 2002, Faries et al., 2005, Ganguly et al., 2004, Ito et al., 2005, Jaffe and Levine, 2003, Janssen et al., 2005, Misawa et al., 2011, Sim et al., 2004, Suokas et al., 2012, Taylor et al., 2002) and has often included polypharmacy during cross-titration, which has hampered definitive conclusions on predictors. More recently research has begun to investigate APP with longer duration (> 60 days) in an attempt to distinguish between cross-titration and long-term treatment (Barbui et al., 2006, Faries et al., 2005, Ganguly et al., 2004, Kadra et al., 2015, Kreyenbuhl et al., 2007a, Kreyenbuhl et al., 2007b, Morrato et al., 2007). However, cross-titration is a process that can take up to 10 weeks to complete (Correll et al., 2011, Lochmann van Bennekom et al., 2013); therefore studies examining APP with a duration of 70 days or less cannot definitively exclude switching. Aside from heterogeneity in APP definition, investigations to date have risked residual confounding due to limited covariates in models (Centorrino et al., 2004, Faries et al., 2005). Furthermore, limitations in being able to distinguish temporally between the occurrence of APP prescribing and associated factors, makes it difficult to determine if the latter are predictors or consequences (Kreyenbuhl et al., 2007a, Kreyenbuhl et al., 2007b). Another limitation include small homogenous inpatient samples (Centorrino et al., 2004, Centorrino et al., 2005), restricting generalisability.

Using data derived from a large de-identified electronic health records case register with near-universal coverage of a defined population, we investigated socio-demographic, socioeconomic, clinical, and service-use predictors of long-term APP initiation in SMI.

## 2 Materials and methods

### 2.1 Study design, data source and study sample

This was a retrospective cohort study within a comprehensive register of patients treated with SMI in the South London and Maudsley (SLaM) NHS Foundation Trust. SLaM is one of the largest secondary mental health care providers in Europe, serving four London boroughs (Lambeth, Southwark, Lewisham and Croydon) and around 1.2M residents. As part of the National Health Service, SLaM is the universal provider of mental health services to this population. The Clinical Record Interactive Search (CRIS) was developed in 2008 and enables researchers to search and retrieve de-identified electronic health records (EHRs) information for over 250,000 service users in SLaM. The CRIS system has been described in detail (Perera et al., 2016, Stewart et al., 2009) and is approved by the Oxfordshire Research Ethics Committee C (reference 08/H606/71 + 5) as a database for secondary analysis.

Using CRIS, we ascertained all adult patients with a diagnosis of schizophrenia (ICD-10 code: F20.x), schizoaffective disorder (F25.x) or bipolar disorder (F31.x) and who were in contact with SLaM clinical services between 1st July 2011 and 30th June 2012. All potential predictors were measured prior to 1st January 2012. APP initiation was determined for the period between 1st January and 30th June 2012, also referred to as the follow-up period.

### 2.2 Outcome measures

The primary outcome was long-term antipsychotic polypharmacy (APP) initiation, defined as the concomitant prescription of two or more antipsychotic agents for at least six months, with the aim of minimising the likelihood of cross-titration. All service users who commenced APP at some point from 1st January to 30th June 2012, but who had not received APP in the 6 months prior to this were considered to have been ‘initiated’ on long term APP. A detailed account of the method used for APP ascertaining in CRIS and the validation of this technique have been described elsewhere (Kadra et al., 2015).

### 2.3 Explanatory variables

Age was calculated on the 1st January 2012 and categorised by quartiles. The remaining socio-demographic and socioeconomic factors were derived from the last entry recorded prior to 1st January 2012. Seventeen ethnic group categories were collapsed into “White”, “Black Caribbean”, “Black African” and “Other”, due to small numbers in some cells. Relationship status was defined as “in a current relationship” (cohabitating, married or civil partnership) and “not in a relationship” (single, divorced, separated, widowed, unknown). Employment status was recorded as being in “paid employment” (part-time, full-time, self-employed) and “not in paid employment” (unemployed, registered disabled, retired, student, looking after children, volunteer, in training, not known, other). We used an area-level index of multiple deprivation to estimate socioeconomic status based on seven domains of deprivation ascertained from 2007 UK Census estimates (employment, income, education, health, barriers to housing and services, crime, and living environment), which are weighted and combined into an overall score applied to a given geographic area (DCLG, 2011). In this case, multiple deprivation indices were applied to lower super output areas (LSOAs), which are the smallest enumeration unit, each containing on average 1500 residents (DCLG, 2011). LSOAs were categorised in tertiles based on the four catchment boroughs. In addition, homelessness (Noble et al., 2008) was ascertained based on ‘no fixed abode’ codes.

Clinical symptom presence/severity was estimated from the most recent Health of the Nation Outcome Scale (HoNOS) completed prior to 1st January 2012. HoNOS is a clinical outcome instrument in wide routine use, composed of 12 items designed to measure behaviour, impairment, symptoms, and social functioning (Wing et al., 1998). Items are scored on a scale of 0 (no problem) to 4 (severe to very severe problem). Due to small cell sizes, subscale scores were collapsed into three categories: 0 “not a problem”; 1 “minor problem requiring no action”; 2–4 “significant problem” (Hayes et al., 2012). Items that provided overlapping information to other variables used in this analyses were removed; therefore we did not include item 9 (assessing relationship problems), item 11 (assessing living conditions) and item 12 (assessing occupational problems). Item 8 (assessing other mental health problems) was also excluded, as the following comorbid diagnoses were ascertained using information available from free-text and structured fields: i) depression [having received a diagnosis of depression (ICD-10: F32, F33) and/or scoring ‘mild’ to ‘significant’ on HoNOS item 7]; ii) substance use [having received a diagnosis of substance use disorder (ICD-10: F10–16) and/or scoring ‘mild’ to ‘significant’ on HoNOS item 3]; and iii) personality disorder [having received a diagnosis of personality disorder (ICD-10: F60; F61)].

We considered six measures of service use: i) previous outpatient contact was determined through the proportion of days each person had received face-to-face contact as an outpatient between 1st July and 31st December 2011 (multiple events on a single day were counted as one day of clinical contact, whilst clinical contact with outpatient services during an inpatient admission was not counted); ii) the number of days spent as an in-patient between 1st July and 31st December 2011 were determined separately; iii) we identified the number of previous antipsychotics used in the six months prior to follow-up; iv) we identified all patients who had received a community treatment order (CTO) prior to the start of follow-up [CTOs refer to a conditional discharge from inpatient admission, commonly implemented for a period of six months to improve adherence to medication and promote regular contact with services (DoH, 2007)]; dichotomous variables were generated to indicate whether, since 2007, patients v) had ever used clozapine or vi) ever used a long acting injectable (LAI) antipsychotic agent.

### 2.4 Statistical analysis

STATA 12 was used to conduct all statistical analyses. We estimated APP prevalence and incidence of newly initiated long-term APP in a six-month window. Further analyses focused on predictors of long-term polypharmacy initiation. Multivariable models included potential confounders such as age, gender, ethnicity, relationship status, employment, and deprivation status. Clinical and service use factors were not included as covariates due to possible over-adjustment for potential causal pathway factors.

Several sensitivity analyses were carried out. Firstly, we tested whether the timing of the HoNOS assessment had an effect on the association between clinical items and APP initiation, by restricting the analyses to HoNOS scores obtained within the last year prior to the start of follow-up. We further tested whether being a local resident (as opposed to patients referred from the wider national catchment area) had an effect on the association between APP and all exposure variables. Patients resident outside the local catchment area can be referred to SLaM services for specialist treatment, due to particularly severe or treatment-resistant symptoms. Therefore, this group could be inherently different to local patients.

## 3 Results

We identified 7201 adults with a SMI diagnosis who were receiving SLaM care between January and June 2012. We excluded 344 patients as they were not receiving care in SLaM services in the six months prior to 1st January 2012, resulting in a total sample size of 6857 patients. We found that 331 (4.8%) patients were receiving antipsychotic polypharmacy for six or more months between 1st January and 30th June 2012 (this sample is also referred to as overall APP) and 115 (1.7%) were newly initiated on long-term APP. Table 1 summarises the characteristics for the total cohort and by overall and newly prescribed APP.

Table 2 describes the prevalence of first (FGA) and second-generation antipsychotics (SGA) that were prescribed as part of APP. Two or more SGAs were most commonly co-prescribed. Of the newly initiated sample, 24.3% were receiving clozapine APP.

Table 3 summarises results from the unadjusted and adjusted logistic regression models, which examine the potential socio-demographic and socioeconomic predictors of newly prescribed APP. In the fully adjusted model, individuals in early adulthood (aged 16–35) were more likely to be initiated on APP than older adults (aged 56 +) (OR 2.1, 95% CI 1.1–3.7, p = 0.016), whereas being in a relationship was associated with a reduced risk for APP initiation (OR 0.3, 0.1–0.9, p = 0.043). Experiencing a high level of deprivation and more specifically being homeless was also associated with an increased risk for being newly initiated on long-term APP (OR 3.3, 1.1–9.9, p = 0031).

As described in Table 4, overall clinical symptoms, as measured by HoNOS administered closest to the start of follow-up, were not predictive of APP initiation, with the exception of significant problems with hallucinations and/or delusions (OR 1.6, 1.0–2.5, p = 0.048). In a sensitivity analysis, where the investigation was restricted to HoNOS scores obtained within the last year prior to the observation period, this association was not substantially changed in strength, although fell outside statistical significance (OR 1.5, 0.9–2.4, p = 0.146).

Table 5 summarises associations between newly prescribed APP and service use. We found that the risk of APP initiation increased with every additional day of outpatient contact (OR 1.0099, 1.0002–1.0197, p = 0.045) received in the previous six months, even after adjusting for possible confounders. Similarly, having previously received a CTO (OR 2.6, 1.5–4.5, p < 0.001), previous use of clozapine (OR 1.8, 1.2–2.7, p = 0.006), and previous LAI use (OR 2.2, 1.5–3.2, p < 0.001) were all associated with increased risk of being newly prescribed long-term APP in the fully adjusted models.

In total, 419 (6.7%) patients in the sample had been referred for SLAM services from other boroughs rather than being catchment area residents. A sensitivity analysis indicated that after restricting the analyses to patients residing in the SLaM catchment area, the magnitude and direction of ORs were similar for all associations; however some were no longer significant including being in a relationship (p = 0.056), having problems with hallucinations and/or delusions (p = 0.123) and outpatient contact in the previous 6 months (p = 0.058). Also, after excluding patients from outside the catchment there were no longer any homeless people prescribed long-term APP; therefore an analysis of this variable was not possible.

## 4 Discussion

Our results indicate that age, socioeconomic circumstances, psychotic symptoms, prior outpatient contact, CTOs, prior clozapine and/or LAI use are significant, independent predictors of newly prescribed long-term APP.

Our findings are in keeping with existing research (Mace and Taylor, 2015) indicating that SLaM has a considerably lower prevalence of APP in comparison to a UK national sample and other US studies (Freudenreich and Goff, 2002, Gallego et al., 2012). Considering service use measures, our results both confirm previous research and generate novel findings. For example, our results support previous research which has indicated that prior service use, such as more frequent outpatient contact (Ganguly et al., 2004, Kreyenbuhl et al., 2007a, Kreyenbuhl et al., 2007b), previous use of LAI, and clozapine (Ganguly et al., 2004), are associated with an increased risk for longer term APP (i.e. > 60 days). Importantly, our findings further indicate that only a third of the patients initiated on APP had previously been trialled on clozapine. This has been previously suggested (Howes et al., 2012, Nielsen et al., 2012), and highlights that prescribing guidelines (i.e. that APP should only be considered after trials of two individual agents followed by clozapine) are not consistently applied in ‘real world’ practice. Contrary to some previous reports, we found no evidence to suggest that APP initiation is predicted by the number of days spent as an inpatient or number of antipsychotics used (Barbui et al., 2006, Ganguly et al., 2004, Morrato et al., 2007) in the previous six months. An important issue to bear in mind is that we specifically investigated APP initiation, while previous research has rarely been able to account for pre-existing APP use and thus has not been able to distinguish factors associated with its initiation from those associated with its continuation. Furthermore, it is important to consider service use predictors in the context of the service where they are examined. For example, in the UK, there has been a nationwide drive to reduce the number and duration of inpatient admissions. Therefore, it is possible that factors, which would have previously warranted an inpatient admission, are now possibly driving APP prescribing due to limited beds. Future studies may benefit from testing further whether APP is initiated in the community in order to prevent hospital admission. Our results further suggest that factors such as prior history of CTOs (a proposed proxy for non-adherence) are associated with an increased risk for long-term APP, something that has not been previously investigated (Biancosino et al., 2005, Patel et al., 2011).

Experiencing significant hallucinations and/or delusions, as rated on the respective HoNOS sub-scale, emerged as the sole symptomatic predictor of long-term APP initiation. This contrasts with some previous studies, where no associations were found between general psychopathology and long-term APP (Barbui et al., 2006); however, most studies of smaller inpatient samples (Biancosino et al., 2005, Centorrino et al., 2004, Centorrino et al., 2005) have indicated an association between APP and positive symptoms. Lastly, despite some previous evidence indicating that comorbid diagnoses such as personality disorder (Ganguly et al., 2004) and depression (Kreyenbuhl et al., 2007a, Kreyenbuhl et al., 2007b) are associated with reduced likelihood of APP prescribing, we detected no such associations with APP initiation.

Of the demographic factors that we examined, we found a positive association between APP and younger age (Kreyenbuhl et al., 2007a, Kreyenbuhl et al., 2007b, Morrato et al., 2007, Suokas et al., 2012). There are several potential explanations. For example, it is possible that younger patients are seen as better able to tolerate side-effects associated with APP (Alexopoulos et al., 2004, Shin et al., 2013) or that higher perceived risk (e.g. of violence) influences prescribing behaviour. Ethnic background and gender, in contrast to other studies (Ganguly et al., 2004, Kreyenbuhl et al., 2007a, Kreyenbuhl et al., 2007b, Suokas et al., 2012), were not significantly associated with APP. We found a potentially protective effect of being in a relationship (Kreyenbuhl et al., 2007a, Kreyenbuhl et al., 2007b), which could suggest that being able to sustain an intimate relationship may be seen as a marker for better functioning and less impairment. Deprivation level emerged as the sole socioeconomic factor that predicted initiating long-term APP. In contrast with previous research where the principal focus has been on employment status (Barbui et al., 2006), our study suggests that deprivation is potentially a more meaningful measure of socioeconomic status. It is possible that homelessness acts as a proxy for illness severity (Gaebel and Zielasek, 2015); however, this association is novel, and the role of socioeconomic features in general warrants further investigation.

This study had several strengths. Measuring predictors prior to APP initiation allowed us to separate the exposures and outcome, thereby reducing the influence of reverse causality. We also examined APP of at least six months duration, which is likely to have excluded cross-titration, although it is possible that some instances may have begun with this (i.e. where a cross-titration was commenced but not completed due to worsening symptoms, resulting in the observed APP). We explored multiple factors simultaneously as predictors and confounders, and used data from a large sample including both inpatients and outpatients. Finally, in common with most NHS Mental Health Trusts in the UK, SLaM is close to being a monopoly mental healthcare provider for its geographic catchment; therefore our sample is likely to be representative of patients seen by secondary care (Stewart et al., 2009).

There were several potential limitations. Despite adjusting for multiple confounders, it is possible that some residual confounding may have occurred. We were unable to measure factors such as duration of illness or stages of treatment as patients entered the observation period. In addition, we were unable to measure clinician related factors such as prescriber experience of initiating APP and knowledge of side-effects and adverse outcomes (Correll and Gallego, 2012, Correll et al., 2011, Gee et al., 2014). In contrast to previous research where standardised symptomatic assessments have been used (e.g. PANSS, BPRS), symptom assessment in this study was limited to individual HoNOS items, measured at one point in time. This scale has received some previous criticism with regards to its measurement of symptoms (Bebbington et al., 1999, Stein, 1999), and we were only able to analyse a composite measure of psychotic symptoms. It is possible that true associations may have been concealed, and further research is required into the role of observed and recorded symptomatology in clinical decision-making.

We believe that our findings have several important clinical implications. Long-term APP prescribing is unlikely to be predicted by a single factor, rather it is precipitated by a complex interplay between patient and wider environmental contexts, where clinical symptoms as well as service use such as previous treatment and contact with services may influence decision-making. Furthermore, our study highlights that there are certain patient groups, such as patients whose symptoms are resistant to treatment, that are at an increased risk for APP initiation. Although a proportion of patients prescribed APP do receive pharmacotherapy that is in line with current treatment guidelines (i.e. LAI and clozapine trials that precede APP initiation), a subgroup is offered APP sooner than recommended. Future research would benefit from focusing further on patients that are inappropriately initiated on APP, as a long-term treatment plan. Identifying these groups could encourage clinicians to employ a broader range of interventions, including earlier trials of clozapine and/or alternative treatments to pharmacotherapy to reduce the risk of APP prescribing.

## Role of funding source

This work was supported by the Clinical Records Interactive Search (CRIS) system funded and developed by the National Institute for Health Research (NIHR) Mental Health Biomedical Research Centre at South London and Maudsley NHS Foundation Trust and King's College London and a joint infrastructure grant from Guy's and St Thomas' Charity and the Maudsley Charity (grant number BRC-2011-10035). RDH is funded by a Medical Research Council (MRC) Population Health Scientist Fellowship (grant number MR/J01219X/1). JD is supported by an MRC Clinical Research Training Fellowship MR/L017105/1. All other authors receive salary support from the National Institute for Health Research (NIHR) Mental Health Biomedical Research Centre at South London and Maudsley NHS Foundation Trust and King's College London. The views expressed are those of the author(s) and not necessarily those of the NHS, the NIHR or the Department of Health. The above funding had no role in the study design; in the collection, analysis and interpretation of the data; in the writing of the report; and in the decision to submit the paper for publication.

## Contributors

GK, RDH, and RS designed the study. GK and HS extracted the data and conducted the analysis. All authors contributed to and have approved the final manuscript.

## Conflict of interest

RDH, HS, and RS have received research funding from Roche, Pfizer, J&J and Lundbeck. DT has received research funding from BMS, Janssen and Lundbeck. DT is an Advisory Board member in Lundbeck, Servier and Sunovion.

## Figures and Tables

**Table 1:** Cohort characteristics.

Variables	Total cohort N (%)	APP^a^ N (%)	APP initiation^a^ N (%)
**Total sample**	6857	331 (4.8)	115 (1.7)
**Socio-demographic and socioeconomic factors**			
*Age*			
16–35	1737 (25.3)	117 (35.3)	45 (39.1)
36–45	1789 (26.1)	105 (31.7)	31 (27.0)
46–55	1678 (24.5)	77 (23.3)	22 (19.1)
56 +	1653 (24.1)	32 (9.7)	17 (14.8)
*Gender*			
Female	2821 (41.1)	111 (33.5)	40 (34.8)
Male	4036 (58.9)	220 (66.5)	75 (65.2)
*Ethnicity group*			
White	3124 (45.6)	125 (37.8)	41 (35.7)
Black Caribbean	992 (14.5)	54 (16.3)	16 (13.9)
Black African	1851 (26.9)	106 (32.0)	43 (37.4)
Other	890 (13.0)	46 (13.9)	15 (13.0)
*Relationship status*			
Not in a relationship	6052 (88.3)	311 (94.0)	111 (96.5)
In relationship	805 (11.7)	20 (6.0)	4 (3.5)
*Employment status*			
Not in paid employment	6521 (95.1)	326 (98.5)	113 (98.3)
In paid employment	336 (4.9)	5 (1.5)	2 (1.7)
*Deprivation level in area of residence*			
Low level	2087 (32.7)	95 (30.2)	25 (22.5)
Medium level	2111 (33.1)	113 (35.9)	40 (36.1)
High level	2107 (33.0)	97 (30.8)	42 (37.8)
Homelessness	74 (1.2)	10 (3.1)	4 (3.6)
**Clinical factors**			
*Comorbid diagnosis*			
Depression	3437 (50.1)	165 (49.9)	50 (43.5)
Personality disorder	895 (13.1)	63 (19.0)	20 (17.4)
Substance use	1956 (28.5)	103 (31.1)	38 (33.0)
*Overactive and aggressive behaviour*			
Not a problem	4127 (64.9)	198 (62.3)	64 (59.3)
Minor problem	1333 (21.0)	66 (20.8)	26 (24.0)
Significant problem	898 (14.1)	54 (16.9)	18 (16.7)
*Non-accidental self-injury*			
Not a problem	5850 (92.1)	292 (91.8)	100 (92.6)
Minor problem	326 (5.1)	20 (6.3)	5 (4.6)
Significant problem	179 (2.8)	6 (1.9)	3 (2.8)
*Cognitive problems*			
Not a problem	3799 (59.9)	181 (57.3)	64 (59.8)
Minor problem	1578 (24.9)	83 (26.3)	23 (21.5)
Significant problem	966 (15.2)	52 (16.4)	20 (18.7)
*Physical illness or disability*			
Not a problem	3502 (55.2)	175 (55.0)	57 (52.8)
Minor problem	1254 (19.8)	73 (23.0)	23 (21.3)
Significant problem	1591 (25.0)	70 (22.0)	28 (25.9)
*Hallucinations and delusions*			
Not a problem	2688 (42.3)	97 (30.5)	34 (31.5)
Minor problem	1314 (20.7)	74 (23.3)	25 (23.1)
Significant problem	2348 (37.0)	147 (46.2)	49 (45.4)
*Problems with activities of daily living*			
Not a problem	2842 (44.8)	121 (38.2)	44 (41.1)
Minor problem	1572 (24.8)	86 (27.1)	28 (26.2)
Significant problem	1934 (30.4)	110 (34.7)	35 (32.7)
**Service use**			
*Days of inpatients stay in previous six months,mean* ± *SD (range)*	11.8 ± 36.3 (0–184)	35.8 ± 60.0 (0–184)	18.8 ± 44.2 (0–184)
*Days of outpatient contact in previous six months,mean* ± *SD (range)*	9.4 ± 13.9 (0–174)	12.5 ± 15.1 (0–153)	12.2 ± 20.1 (0–153)
*Previous CTOs*			
No	6483 (94.6)	394 (88.8)	98 (85.2)
Yes	374 (5.4)	37 (11.2)	17 (14.8)
*Number of antipsychotics used in the previous six months,mean* ± *SD (range)*	1.0 ± 1.0 (0–8)	2.2 ± 1.2 (0–7)	1.2 ± 1.0 (0–6)
*Previous clozapine use*			
No	5643 (82.3)	151 (45.6)	80 (69.6)
Yes	1214 (17.7)	180 (54.4)	35 (30.4)
*Previous LAI use*			
No	4405 (64.2)	167 (50.5)	52 (45.2)
Yes	2452 (35.8)	164 (49.5)	63 (54.8)

a Antipsychotic polypharmacy (APP) lasting for six or more months.

**Table 2:** Prevalence and distribution of long-term antipsychotic polypharmacy (APP).

Types of antipsychotic polypharmacy	APP (n = 331)		APP initiation (n = 115)	
n	% (95% CI)	n	% (95% CI)
First generation antipsychotics (FGA) only	9	2.7 (1.3–5.1)	6	5.2 (1.9–11.0)
Second generation antipsychotics (SGA) only	216	65.3 (59.9–70.4)	62	53.9 (44.4–63.2)
FGA + SGA	106	32.0 (27.0–37.3)	47	40.9 (31.8–50.4)
APP inclusive of FGA or SGA LAI^a^	72	21.8 (17.3–26.2)	35	30.4 (21.9–38.9)
APP inclusive of clozapine^a^	165	49.9 (44.4–55.3)	28	24.3 (16.4–32.3)

a Categories overlap with APP by generation (FGA; SGA; FGA + SGA).

**Table 3:** Logistic regression analysis of socio-demographic and socioeconomic predictors of antipsychotic polypharmacy initiation.

	Crude OR (95% CI)	Adjusted OR^a^ (95% CI)	Adjusted p-value
*Age*			
16–35	**2.6 (1.6–4.5)**	**2.1 (1.1–3.7)**	**0.016**
36–45	1.7 (0.9–3.0)	1.4 (0.8–2.6)	0.291
46–55	1.3 (0.7–2.4)	1.1 (0.6–2.1)	0.749
56 +	Reference	Reference	

*Gender*			
Female	Reference	Reference	
Male	1.3 (0.9–1.9)	1.1 (0.7–1.7)	0.621

*Ethnicity group*			
White	Reference	Reference	
Black Caribbean	1.2 (0.7–2.2)	1.1 (0.6–2.1)	0.691
Black African	**1.8 (1.2–2.8)**	1.4 (0.9–2.3)	0.129
Other	1.3 (0.7–2.3)	1.3 (0.7–2.4)	0.403

*Relationship status*			
Not in a relationship	Reference	Reference	
In relationship	**0.3 (0.1–0.7)**	**0.3 (0.1–0.9)**	**0.043**

*Employment status*			
Not in paid employment	Reference	Reference	
In paid employment	0.3 (0.1–1.4)	0.4 (0.1–1.6)	0.181

*Deprivation level*			
Low level	Reference	Reference	
Medium level	1.6 (0.9–2.6)	1.4 (0.9–2.4)	0.164
High level	**1.7 (1.0–2.8)**	1.5 (0.9–2.5)	0.116
Homelessness	**4.7 (1.6–13.9)**	**3.3 (1.1–9.9)**	**0.031**

Values in bold are statistically significant (≤ 0.05).

a Models adjusted for all socio-demographic and socioeconomic factors.

**Table 4:** Logistic regression analysis of clinical predictors of antipsychotic polypharmacy initiation.

	Crude OR (95%CI)	Adjusted OR^a^ (95% CI)	Adjusted p-value
*Comorbid diagnosis*			
Depression			
No	Reference	Reference	
Yes	0.8 (0.5–1.1)	0.8 (0.6–1.2)	0.286
Personality disorder			
No	Reference	Reference	
Yes	1.4 (0.9–2.3)	1.2 (0.7–2.0)	0.464
Substance use			
No	Reference	Reference	
Yes	1.2 (0.8–1.8)	1.0 (0.6–1.5)	0.873

*Overactive and aggressive behaviour*			
Not a problem	Reference	Reference	
Minor problem	1.3 (0.8–2.0)	1.3 (0.8–2.0)	0.331
Significant problem	1.3 (0.8–2.2)	1.3 (0.8–2.2)	0.339

*Non-accidental self-injury*			
Not a problem	Reference	Reference	
Minor problem	0.9 (0.4–2.2)	0.9 (0.4–2.2)	0.804
Significant problem	1.0 (0.3–3.1)	0.9 (0.3–3.0)	0.922

*Cognitive problems*			
Not a problem	Reference	Reference	
Minor problem	0.9 (0.5–1.4)	0.9 (0.6–1.5)	0.818
Significant problem	1.2 (0.7–2.0)	1.4 (0.8–2.3)	0.222

*Physical illness or disability*			
Not a problem	Reference	Reference	
Minor problem	1.1 (0.7–1.8)	1.4 (0.9–2.4)	0.159
Significant problem	1.1 (0.7–1.7)	1.6 (0.9–2.6)	0.064

*Hallucinations and delusions*			
Not a problem	Reference	Reference	
Minor problem	1.5 (0.9–2.5)	1.5 (0.9–2.5)	0.141
Significant problem	**1.7 (1.1–2.6)**	**1.6 (1.0–2.5)**	**0.048**

*Problems with activities of daily living*			
Not a problem	Reference	Reference	
Minor problem	1.2 (0.7–1.9)	1.2 (0.7–1.9)	0.506
Significant problem	1.2 (0.8–1.8)	1.2 (0.8–1.9)	0.414

Values in bold are statistically significant (≤ 0.05).

a Models adjusted for all socio-demographic and socioeconomic factors.

**Table 5:** Logistic regression analysis of service use predictors of antipsychotic polypharmacy initiation.

	Crude OR (95% CI)	Adjusted OR^a^ (95% CI)	Adjusted p-value
Days of inpatients stay in previous six months	**1.0 (1.00–1.01)**	1.0 (0.9–1.0)	0.309
Days of outpatient contact in previous six months	**1.0095 (1.0007–1.0183)**	**1.0099 (1.0002–1.0197)**	**0.045**
Number of antipsychotics used in the previous six months	**1.2 (1.0–1.4)**	1.1 (0.9–1.3)	0.291
Previous CTOs			
No	Reference	Reference	
Yes	**3.1 (1.8–5.2)**	**2.6 (1.5–4.5)**	**< 0.001**
Previous clozapine use			
No	Reference	Reference	
Yes	**2.1 (1.4–3.1)**	**1.8 (1.2–2.7)**	**0.006**
Previous LAI use			
No	Reference	Reference	
Yes	**2.2 (1.5–3.2)**	**2.2 (1.5–3.2)**	**< 0.001**

Values in bold are statistically significant (≤ 0.05).

a Models adjusted for all socio-demographic and socioeconomic factors.
